# Toxicity evaluation of processing Evodiae fructus based on intestinal microbiota

**DOI:** 10.3389/fmicb.2024.1336777

**Published:** 2024-02-16

**Authors:** Xuejuan Liang, Jing Liu, Jiaxin Di, Nenqun Xiao, Yanmei Peng, Qixue Tian, Linglong Chen

**Affiliations:** ^1^Hunan Academy of Chinese Medicine, Changsha, China; ^2^Hunan University of Chinese Medicine, Changsha, China; ^3^Hunan Province Hospital of Integrated Traditional Chinese and Western Medicine (The Affiliated Hospital of Hunan Academy of Traditional Chinese Medicine), Changsha, China; ^4^National Traditional Chinese Medicine Processing Technology Inheritance Base of the Affiliated Hospital of Hunan Academy of Traditional Chinese Medicine, Changsha, China

**Keywords:** Evodiae fructus, nephrotoxic, hepatotoxicity, intestinal microbiota, toxicity

## Abstract

**Background:**

With the development of healthcare services, drug efficacy, and safety have become the focus of drug use, and processing alters drug toxicity and efficacy, exploring the effects of processing on Evodiae fructus (EF) can guide the clinical use of drugs.

**Methods:**

Fifty male Kunming mice were randomly divided into the control group (CCN), raw small-flowered EF group (CRSEF), raw medium-flowered EF group (CRMEF), processing small-flowered EF group (CPSEF), and processing medium-flowered EF group (CPMEF). The CRSEF, CRMEF, CPSEF, and CPMEF groups were gavaged with aqueous extracts of raw small-flowered EF dry paste (RSEF), medium-flowered EF dry paste (RMEF), processing small-flowered EF dry paste (PSEF) and processing medium-flowered EF dry paste (PMEF), respectively, for 21 days at 5 times the pharmacopeial dosage. Upon concluding the experiment, histopathological sections of liver and kidney tissues were examined. Additionally, levels of aspartate aminotransferase (AST), alanine aminotransferase (ALT), serum creatinine (SCr), and blood urea nitrogen (BUN) were determined. DNA from the intestinal contents of the mice was extracted, and 16S rRNA full-length high-throughput sequencing was performed.

**Results:**

After fed EF 21 days, mice exhibited a decreasing trend in body weight. Comparative analysis with the CCN group revealed an upward trend in SCr, BUN, AST, and ALT levels in both CRSEF and CRMEF groups. The CRMEF group displayed notably elevated BUN and AST levels, with an observed increasing trend in Scr and ALT. Kidney sections unveiled cellular edema and considerable inflammatory cell infiltrates, whereas significant liver damage was not evident. Compared with CRSEF, Bun levels were significantly lower while AST levels were significantly higher in the CPMEF group. Additionally, the intestinal microbiota diversity and the relative abundance of *Psychrobacter* decreased significantly, and the relative abundance of *Staphylococcus*, *Jeotgalicoccus*, and *Salinicoccus* increased significantly in the CPMEF group. AST, ALT, and SCr were positively correlated with *Staphylococcus*, *Jeotgalicoccus*, and *Salinicoccus*.

**Conclusion:**

In conclusion, PMEF significantly increased harmful bacteria (*Staphylococcus*, *Jeotgalicoccus*, and *Salinicoccu*) and decreased beneficial bacteria. SEF with 5 times the clinical dose showed nephrotoxicity and SEF nephrotoxicity decreased after processing, but EF hepatotoxicity was not significant, which may be due to insufficient dose concentration and time.

## 1 Introduction

Traditional Chinese Medicine (TCM) is widely used because of its low side effects and good curative effect. In recent years, with the development and application of TCM, the safety issues of its medication have become increasingly prominent. As herbal medicine involves the use of natural compounds, its active ingredients are complex and have different degrees of side effects, there have been extensive literature reports that improper use of Chinese herbal medicine can lead to liver and kidney damage (Yang B. et al., 2018; Wu et al., 2019). Evodiae fructus (EF) is the dried, nearly ripe fruit of *Euodia rutaecarpa* (Juss), which is generally classified according to fruit size as small-flowered EF (SEF), and medium-flowered EF (MEF), and is a herb with the effects of dispersing cold and relieving pain, relieving nausea, and reinforcing Yang to prevent diarrhea (National Pharmacopoeia Commission, 2020). The main chemical composition is alkaloids, terpenoids, flavonoids, phenolic acids, steroids, and phenylpropanoids (Li and Wang, 2020). EF alkaloids contain a variety of chemical components particularly evodiamine (EVO), rutaecarpine, and dehydroevodiamine (Chen et al., 2023). EVO is a natural quinolone alkaloid, which has been widely studied for its extensive pharmacological activities such as anti-tumor, anti-Alzheimer’s disease, anti-pulmonary hypertension, anti-fungal and anti-inflammatory (Li D. et al., 2022). According to the 2020 edition of Chinese Pharmacopoeia (National Pharmacopoeia Commission, 2020), EF mainly enters the liver, spleen, and kidney meridians. Modern studies have proved that EF was mainly used for the treatment and prevention of gastrointestinal diseases (Tan and Zhang, 2016), and can treat diarrhea of kidney-yang deficiency syndrome. The kidney structure, energy metabolism, and intestinal mucosal microbiota diversity and structure were improved (Zhu et al., 2023).

Some studies have shown significant hepatotoxicity and nephrotoxicity of EF. Compounds of EF can increase the activity of aspartate aminotransferase (AST), alanine aminotransferase (ALT), lactate dehydrogenase (LDH), and alkaline phosphatase (ALP) with potential liver damaging effects (Li et al., 2019). EF hepatotoxicity is mainly associated with oxidative stress, mitochondrial damage and dysfunction, endoplasmic reticulum stress, hepatic metabolic disorders, and apoptosis (Yan et al., 2023). In terms of nephrotoxicity, the LDH release, renal cell morphology changes, and autophagy-related protein expression of cells treated with EVO at different concentrations increased, suggesting that EVO renal cytotoxicity may be associated with cellular autophagy-related pathways (Yan et al., 2017). The Chinese Pharmacopoeia (National Pharmacopoeia Commission, 2020) recorded that EF has a small toxicity. Therefore, improper use of EF not only fails to achieve therapeutic effect but will cause body damage.

The composition of the intestinal microbiota is susceptible to dietary and pharmacological influences and can have both beneficial and detrimental effects on the host organism (Li C. et al., 2022). It has been found that EF has a good modulating effect on intestinal microbiota, and changes in intestinal microbiota can respond to changes in intestinal function. Studies have found that adhesion and biofilm formation are key factors in the persistence of *Campylobacter jejuni* under adverse environmental conditions and that EF can weaken adhesion and inhibit the growth of harmful bacteria (Bezek et al., 2016). EVO can balance the levels of *Escherichia coli* and *Lactobacillus* levels to reduce intestinal inflammation (Shen et al., 2019). EVO also promotes enrichment, reduces intestinal inflammation, promotes the enrichment of Short-chain fatty acids (SCFAs) producing bacteria, reduces pro-inflammatory bacteria, regulates intestinal microbiota metabolites, and inhibits the development of intestinal inflammation (Wang et al., 2021). Therefore, appropriate intake of EF can change the intestinal microbiota and thus affect the health of the organism. However, the effects of raw EF and processing EF on the intestinal microecological mechanisms are unclear, and there are few studies on the effects of processed factors on the regulation of EF on the intestinal microbiota. A large number of studies have found that disorders of the intestinal microbiota are closely related to the development of disease. The microbiota regulates the immune and hormone levels of the liver or kidneys by sending signals to distant organs and tissues in the body. The interaction between the intestinal and the kidney and the liver is called the “liver-gut axis” and the “kidney-gut axis” (Schroeder and Bäckhed, 2016; Yang T. et al., 2018). Based on the above, we hypothesize that EF may regulate the metabolism of the liver, kidney, and intestinal microbiota through the “liver-gut axis” and “kidney-gut axis.”

A summary of relevant literature found that EF exhibited significant time-and dose-dependent hepatotoxicity (Li and Sun, 2015). We hypothesized that EF toxicity is mainly related to the concentration and time of administration, and processing can reduce the toxicity of the drug and change its properties of the drug. At present, the study of EF is limited to the extraction of the active ingredient, but the study of toxic concentration and processing methods to reduce toxicity is not clear.

## 2 Materials

### 2.1 Medicines and feed

Raw small-flowered EF and raw medium-flowered EF: purifying processing. 1.8 kg of liquorice is boiled to produce 12.6 kg of liquorice juice, which is then combined with 30 kg of raw EF. After thorough mixing and absorption of the liquorice juice, the mixture is gently stir-heated until dry, resulting in the creation of processed small-flowered EF and processed medium-flowered EF (Zhang et al., 2018). One kg samples of the above four EF variants are individually weighed, combined with 12 times their respective water content, and subjected to three reflux extractions, each lasting 40 min. The two filtrates were combined and concentrated to an infusion with a relative density of 1.23 (tested at 60°C) and dried under vacuum at 55°C to form an aqueous extract of raw small-flowered EF dry paste (RSEF), aqueous extract of raw medium-flowered EF dry paste (RMEF), aqueous extract of processing small-flowered EF dry paste (PSEF), and aqueous extract of processing medium-flowered EF dry paste (PMEF). Table 1 provides details on the yield of the four aqueous EF dry paste extracts, along with the content of Limonin and EVO in each.

**Table 1:** The yield rate and the contents of Limonin and EVO in aqueous extracts of different EF.

Sample name	RSEF	RMEF	PSEF	PMEF
Dried paste folded raw medicine amount (1:1)	3.5721	2.4040	2.0613	2.7686
Limonin Content in dry paste (mg/g)	15.76	18.97	8.84	15.46
EVO content in dry paste (mg/g)	0.67	0.78	0.43	0.91

RSEF: aqueous extract of raw small-flowered EF dry paste, RMEF: aqueous extract of raw medium-flowered EF dry paste, PSEF: aqueous extract of processing small-flowered EF dry paste, PMEF: aqueous extract of processing medium-flowered EF dry paste.

The experimental animal common feed, comprising crude protein, crude fiber, crude fat, crude ash, calcium, phosphorus, lysine, methionine, cysteine, and so on, was sourced from the Experimental Animal Center of Hunan University of Chinese Medicine and manufactured by Jiangsu Medison Biomedical Co., Ltd.

### 2.2 Animal

In this study, to exclude the effect of sex on gut microbiota (Wu et al., 2022), 50 SPF-grade Kunming male mice (20 ± 2 g) were used. The animals were provided by Hunan Slaughter Jingda Laboratory Animal Co., Ltd. (license number: SCXK2019-0004), and fed at the Animal Experiment Center of Hunan University of Chinese Medicine. All animal operations were performed by the Guide for the Care and Use of Laboratory Animals of the Hunan University of Chinese Medicine and were approved by the Animal Experimentation Ethics Committee of the Hunan University of Chinese Medicine (ethics number: LL2022112302).

## 3 Methods

### 3.1 Preparation of EF solution

It was found that the toxicity of EF gradually increased with the increase of drug concentration and time, and hepatotoxicity appeared when the dose was five times the clinical equivalent dose (Zhang et al., 2018). Based on the Chinese Pharmacopoeia (National Pharmacopoeia Commission, 2020) at the usual clinical dosage of EF (5 g/d for 70 kg adults) and reference (Zhang et al., 2018), the toxicological effect of EF was studied at 5 times the pharmacopeial dosage (25 g/d). The daily doses of EF aqueous extracts were converted to mice based on the yield of different EF aqueous extracts and the body surface area of mice, and the converted doses were 0.91 g/(kg.d) RSEF, 1.35 g/(kg.d) RMEF, 1.58 g/(kg.d) PSEF, and 1.17 g/(kg.d) PMEF for mice. An appropriate amount of water extract of EF dry paste with distilled water was prepared into different concentrations of EF solution.

### 3.2 Animal grouping and drug administration

**Figure 1:**
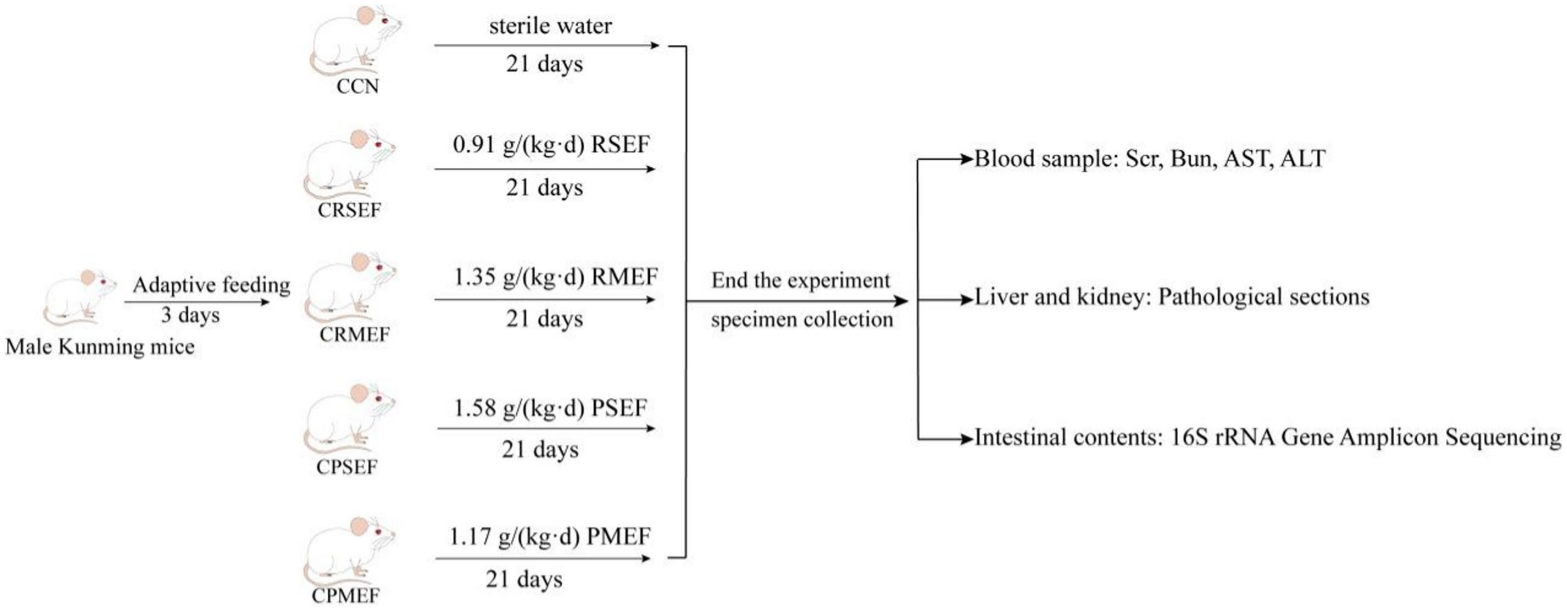
Experimental flow chart. CCN: control group, CRSEF: raw small-flowered EF group, CRMEF: raw medium-flowered EF group, CPSEF: processing small-flowered EF group, CPMEF: processing medium-flowered EF group. RSEF: aqueous extract of raw small-flowered EF dry paste, RMEF: aqueous extract of raw medium-flowered EF dry paste, PSEF: aqueous extract of processing small-flowered EF dry paste, PMEF: aqueous extract of processing medium-flowered EF dry paste.

After adaptive feeding, 50 SPF-grade KM male mice were randomly divided into control group (CCN), raw small-flowered EF group (CRSEF), raw medium-flowered EF group (CRMEF), processing small-flowered EF group (CPSEF), and processing medium-flowered EF group (CPMEF). The CRSEF, CRMEF, CPSEF and CPMEF groups were gavaged with 0.91 g/(kg.d) raw small-flowered EF (RSEF), 1.35 g/(kg.d) raw medium-flowered EF (RMEF), 1.58 g/(kg.d) processing small-flowered EF (PSEF) and 1.17 g/(kg.d) processing medium-flowered EF (PMEF), 0.35 mL at a time, twice a day for 21 days. The CCN group was given sterile water gavage in equal frequency during the period. During the experiment, the mental state, activity level, hair color changes and diet of the mice were mainly observed, and the weight of the mice was recorded, as shown in Figure 1.

### 3.3 Biochemical index testing

After 12 h of fasting after the last dose, whole blood was collected from the eye. The blood stood at room temperature for 2 h, centrifuged at 3,000 r/min for 10 min, and serum was taken for use. According to the instructions of the ELISA test kit, add samples, add antibodies, incubate, wash plates, develop colors, and terminate the reaction in turn. Finally, use the Rayto RT-6100 enzyme-labeled analyzer to detect the OD values of AST and ALT in serum samples at 450 nm. The determination of Bun and SCr is divided into control tube, standard tube, and sample tube according to the instructions of biochemical kit. After adding distilled water, standard substance, and sample to be detected respectively, SCr adds enzyme solution A and incubates at 37°C for 5 min, then determines the absorbance value, and then adds enzyme solution B and incubates at 37°C for 5 min. Bun is mixed with buffered enzyme solution, and the absorbance is measured after water bath at 37°C for 10 min. Then phenol chromogenic agent and alkaline sodium hypochlorite are added and mixed thoroughly, and the absorbance of each tube is measured after water bath at 37°C for 10 min. According to the formula, the contents of SCr and Bun in serum were calculated, and the standard curve equation was drawn to calculate the contents of AST and ALT in each sample.

### 3.4 Pathological sections

Under aseptic conditions, the livers and kidneys were dissected and removed, and the impurities on the surface of the organs were washed with saline and cut into 1–2 cm tissue clumps with scalpel, fixed in 4% paraformaldehyde solution and stored at room temperature. According to gradient dehydration, xylene was transparent and paraffin was embedded. After dressing the wax blocks, the wax blocks were cooled on a −20°C frozen table and sliced in a paraffin slicer (thickness 4 μm). The slices were floated in warm water at 40°C in a sheet spreading machine to flatten the tissues, and the tissues were picked up by slides, roasted in an oven at 60°C, and stored at room temperature for later use. The paraffin section samples were soaked in xylene at one time and then transferred to anhydrous ethanol for dewaxing. After dewaxing, the sections were dyed in HE dye solution in dyeing tank, and then added to concentration gradient ethanol and xylene successively for dehydration, and finally sealed with neutral gum (Liu et al., 2023).

### 3.5 Intestinal contents sample collection

After 21 days, the mice were cervically dislocated and executed, and the small intestine was immediately removed by opening the abdominal cavity on an ultra-clean bench. Under aseptic conditions, the intestinal tissue from the pylorus to the ileocecal region was incised longitudinally with sterile scissors and the intestinal contents were removed with sterile forceps and collected from each mouse in an EP tube and stored at −80°C (Li X. et al., 2022).

### 3.6 Total DNA extraction, PCR amplification, and high-throughput sequencing

Total microbial genomic DNA was extracted from each sample tube according to the OMEGA Soil DNA Kit instructions. The quantity and quality of extracted DNA were measured by using a NanoDrop NC2000 spectrophotometer and agarose gel electrophoresis, respectively. PCR amplification of the bacterial 16S rRNA genes V3–V4 region was performed using the forward primer 338F (5′-ACTCCTACGGGAGGCAGCA-3′) and the reverse primer 806R (5′-GGACTACHVGGGTWTCTAAT-3′). Thermal cycling consisted of initial denaturation at 98°C for 5 min, followed by 25 cycles consisting of denaturation at 98°C for 30 s, annealing at 53°C for 30 s, and extension at 72°C for 45 s, with a final extension of 5 min at 72°C. PCR amplicons were purified with Vazyme VAHTSTM DNA Clean Beads and quantified using the Quant-iT PicoGreen dsDNA Assay Kit. After the individual quantification step, amplicons were pooled in equal amounts, and pair-end 2,250 bp sequencing was performed using the Illlumina NovaSeq platform with NovaSeq 6000 SP Reagent Kit at Shanghai Personal Biotechnology Co., Ltd. (Shanghai, China) (Li X. et al., 2022; Qiao et al., 2023).

### 3.7 Bioinformatics

The original sequence data is decoded by the demux plug-in, the primer is excised by cutadapt plug-in, and then the sequence is processed by DADA2 plug-ins, such as quality filtering, denoising, splicing, and chimera removal. Merge the above-mentioned sequences according to 100% sequence similarity, and generate characteristic sequence ASVs and abundance data table. ASV-level alpha diversity indices were calculated using the amplicon sequence variant (ASV) table in QIIME2 and visualized as box plots. ASV-level ranked abundance curves were generated to compare the richness and evenness of ASVs among samples. Beta diversity analysis was performed to investigate the structural variation of microbial communities across samples using Bray-Curtis metrics and visualized via principal coordinate analysis (PCoA) and nonmetric multidimensional scaling (NMDS). A Venn diagram was generated to visualize the shared and unique ASVs among samples or groups using the R package “VennDiagram,” based on the occurrence of ASVs across samples/groups regardless of their relative abundance. Linear discriminant analysis effect size (LEfSe) was performed to detect differentially abundant taxa across groups using the default parameters. The metabolic function of microbial microbiota was analyzed by PICRUSt2 to obtain functional units and then predicted on the KEGG^1^ database to obtain the abundance value of metabolic pathways (Kanehisa and Goto, 2000; Li L. et al., 2022).

### 3.8 Correlation analysis

Pearson’s correlation coefficient was used when the data satisfied normality, and Spearman’s correlation coefficient was used when it did not satisfy normality. SPSS 21.0 statistical software was used to calculate Spearman correlation coefficients and heat maps were plotted in the cloud platform.^2^ The correlation between two variables is expressed by using the correlation coefficient, and the closer the correlation coefficient is to 1 or −1, the greater the correlation between the two elements, and the closer the correlation coefficient is to 0, the more independent the two elements are.

### 3.9 Statistical analysis

The experimental data were expressed as mean ± standard deviation. The SPSS 21.0 statistical package was used for the statistical analysis of all data. Comparisons between multiple groups, satisfying normality and homoscedasticity, were performed by one-way ANOVA, and comparisons between multiple groups were performed by LSD test. The rank sum test was used if normality and homoscedasticity were not satisfied, and Tamhane’s T2 (M) test was used for comparison between multiple groups. *p* < 0.05 indicates a significant difference.

## 4 Results

### 4.1 Effects of different EF on body weight and liver and kidney functions in mice

**Figure 2:**
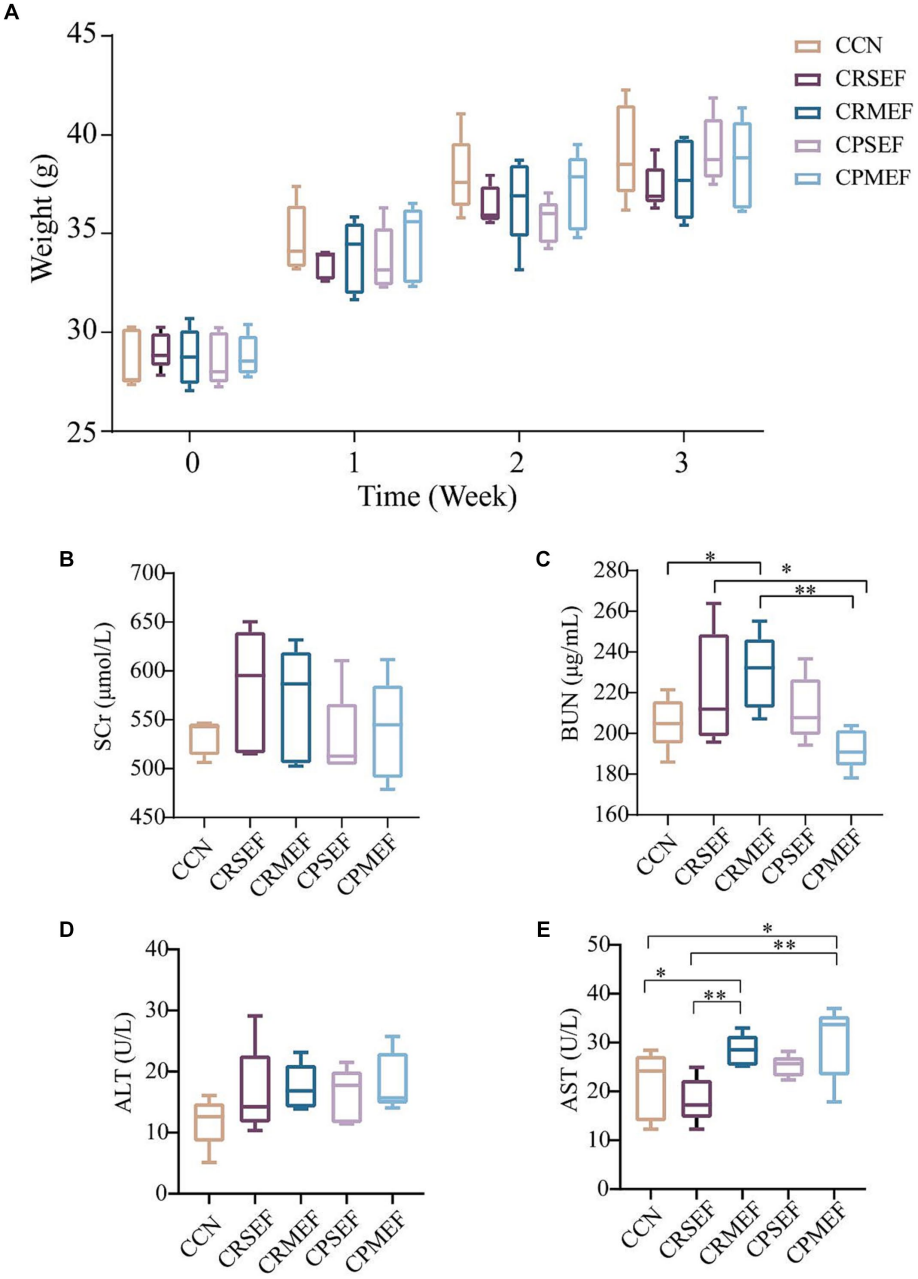
**(A)** Changes in body weight of mice in different groups after 21 days of different EF interventions. **(B)** Serum levels of SCr. **(C)** Serum levels of BUN. **(D)** Serum levels of ALT. **(E)** Serum levels of AST. CCN: control group, CRSEF: raw small-flowered EF group, CRMEF: raw medium-flowered EF group, CPSEF: processing small-flowered EF group, CPMEF: processing medium-flowered EF group. *p < 0.05, **p < 0.01.

Compared with the CCN group, the weight of mice increased slowly after EF intervention (Figure 2A; *p* > 0.05). The main detectors of nephrotoxicity were SCr and BUN. The changes in SCr content were not significant in all groups after EF feeding (Figure 2B; *p* > 0.05). Compared with the CCN group, BUN was significantly higher in the CRMEF group (*p* < 0.05). Compared with the CRMEF group, BUN was significantly lower in the CPMEF group (Figure 2C; *p* < 0.05). The main tests of liver function are AST and ALT. Compared to the CCN group, ALT increased after EF intervention (Figure 2D; *p* > 0.05), and AST in both the CRMEF group and CPMEF group increased significantly (Figure 2E; *p* < 0.05). Compared with CRSEF, AST was significantly higher in the both CRMEF and CPMEF groups (Figure 2E; *p* < 0.05).

### 4.2 Effect of different EF on the histomorphology of the liver and kidney of mice

**Figure 3:**
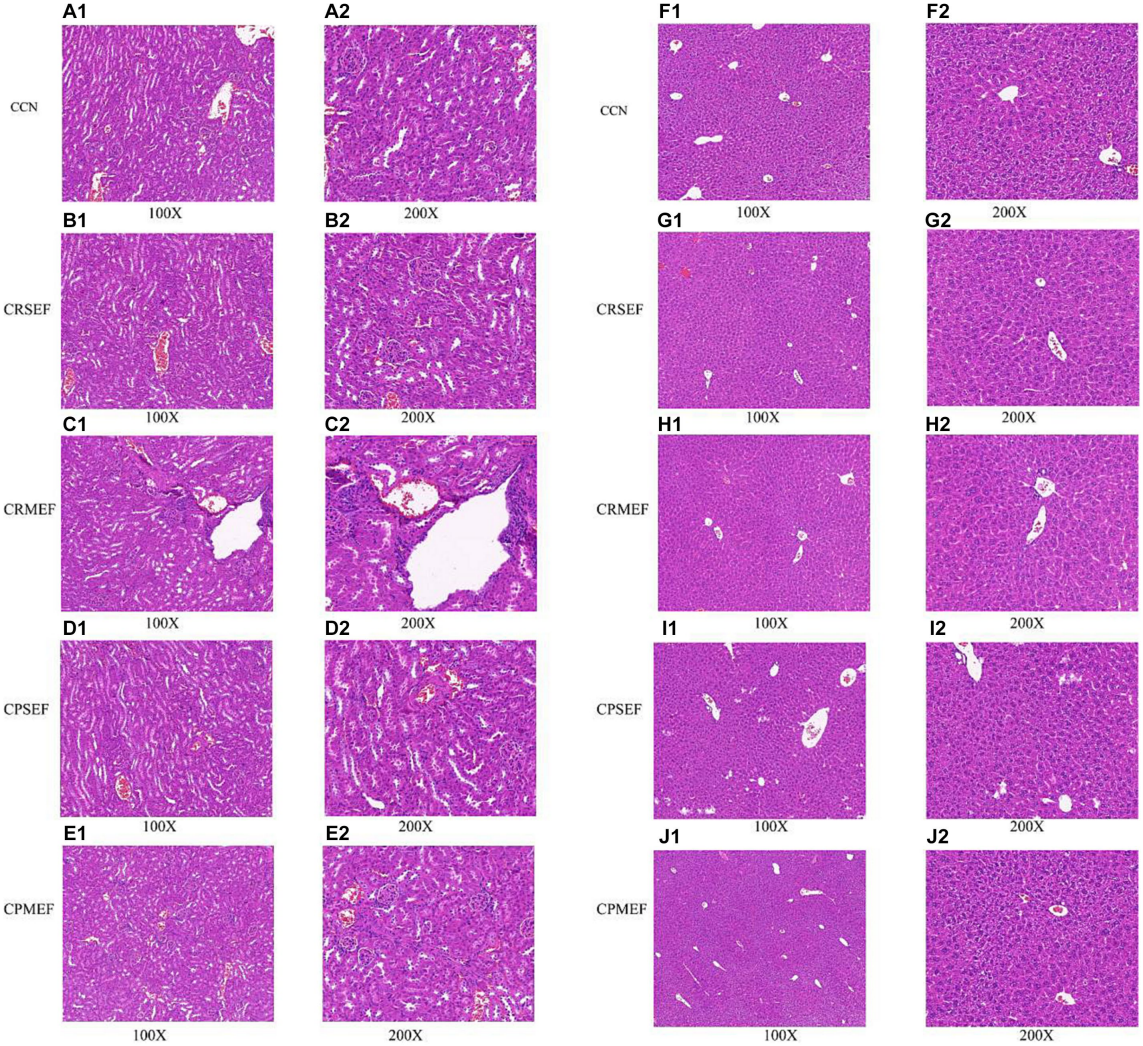
**(A)** Kidney tissue section of CCN group. **(B)** Kidney tissue section of CRSEF group. **(C)** Kidney tissue section of CRMEF group. **(D)** Kidney tissue section of CPSEF group. **(E)** Kidney tissue section of CPMEF group. **(F)** Liver tissue section of CCN group. **(G)** Liver tissue section of CRSEF group. **(H)** Liver tissue section of CRMEF group. **(I)** Liver tissue section of CPSEF group. **(J)** Liver tissue section of CPMEF group. CCN: control group, CRSEF: raw small-flowered EF group, CRMEF: raw medium-flowered EF group, CPSEF: processing small-flowered EF group, CPMEF: processing medium-flowered EF group.

We further evaluated the toxic effects of EF by observing tissue inflammation and injury in combination with histopathological sections of the liver and kidney. The results of light microscopic observation of kidney pathological sections in each group of mice: The kidney tissues of mice in the CCN group had intact peritoneum, clear cortical and medullary demarcation, intact glomerular, tubular, interstitial structures, and uniform distribution (Figure 3A). The renal tubular lumen was heterogeneous with tubular cell edema in both CRSEF and CRMEF groups. A large number of inflammatory cells were infiltrated in the CRMEF group (Figures 3B,C). Glomeruli, tubules, and interstitial structures were intact in both CPSEF and CPMEF groups, with scattered hemorrhages (considering nonspecific injury due to sampling), and partial glomeruli atrophic sclerosis in the CPSEF group (Figures 3D,E).

The results of light microscopic observation of liver pathological sections of mice in each group: In the liver lobules of mice in the CCN group, the hepatocytes were arranged compactly and neatly, with polygonal hepatocytes, pink-stained cytoplasm and rounded nuclei of different sizes, radiating from the central vein (Figure 3F). Scattered blebs were seen in both CRSEF and CRMEF groups (considering nonspecific injury due to sampling), and petechial hemorrhage was present within the CRSEF group (Figures 3G,H). In the CPSEF and CPMEF groups, hepatic lobules with normal hepatocyte morphology and no obvious pathological damage were seen (Figures 3I,J).

### 4.3 Effects of different EF on intestinal contents microbiota of mice

#### 4.3.1 Different EF change the diversity of intestinal contents microbiota in mice

**Figure 4:**
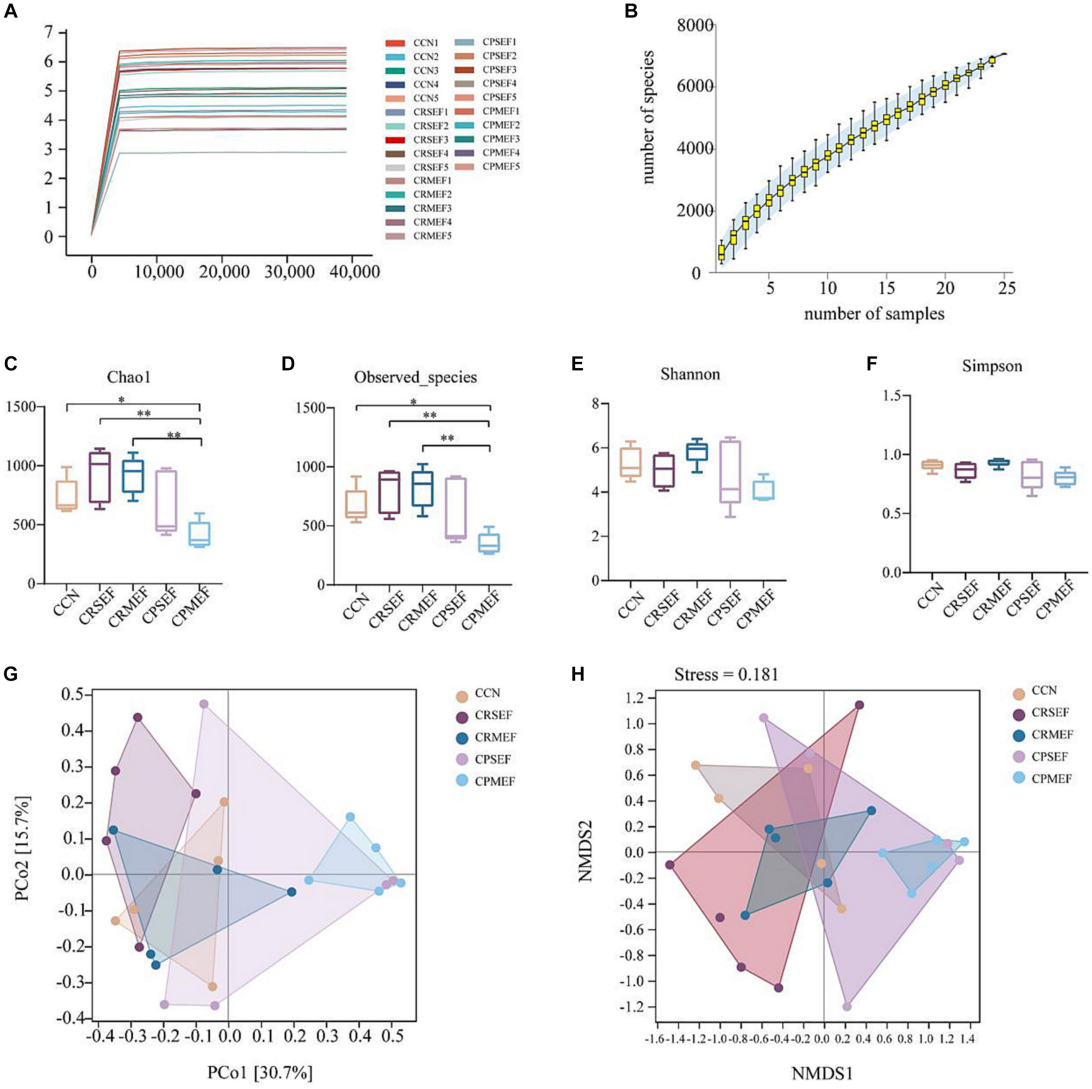
**(A)** Shannon dilution curve for each group of mice. **(B)** Species accumulation curves for each group of mice. Alpha diversity index: **(C)** Chao1 Index. **(D)** Observed species Index. **(E)** Shannon Index. **(F)** Simpson Index. Beta diversity based on Bray-Curtis distance algorithm: **(G)** Distance matrix PCoA analysis. **(H)** Distance matrix NMDS analysis. CCN: control group, CRSEF: raw small-flowered EF group, CRMEF: raw medium-flowered EF group, CPSEF: processing small-flowered EF group, CPMEF: processing medium-flowered EF group. *p < 0.05, **p < 0.01.

As the number of sequencing increased, the growth rate of ASV number decreased, and the Shannon dilution curve gradually flattened, indicating that the sequencing data in this experiment was sufficient (Figure 4A). The species accumulation curve gradually increased with increasing sample size, and the sequencing depth of the experiment was sufficient to reflect the microbial diversity contained in the community samples (Figure 4B). It was found that compared with the CCN group, Chao1 index and Observed species index in the CPMEF group were significantly decreased (*p* < 0.05, Figures 4C,D), and the Simpson and Shannon indices have no significant difference (*p*  > 0.05, Figures 4E,F). Compared with the CPMEF group, Chao1 index and Observed species index in the CRSEF and CRMEF groups were significantly increased (*p* < 0.05, Figures 4C,D). Our study further evaluated beta diversity based on the Bray-Curtis distance algorithm. Distance matrix PCoA and NMDS analysis shows that the CRSEF and CRMEF groups and the CPSEF and CPMEF groups showed better separation at PCo1 = 30.7%, PCo2 = 15.7%, and Stress = 0.181 (Figures 4G,H).

#### 4.3.2 Different EF altered the structure of intestinal contents microbiota in mice

**Figure 5:**
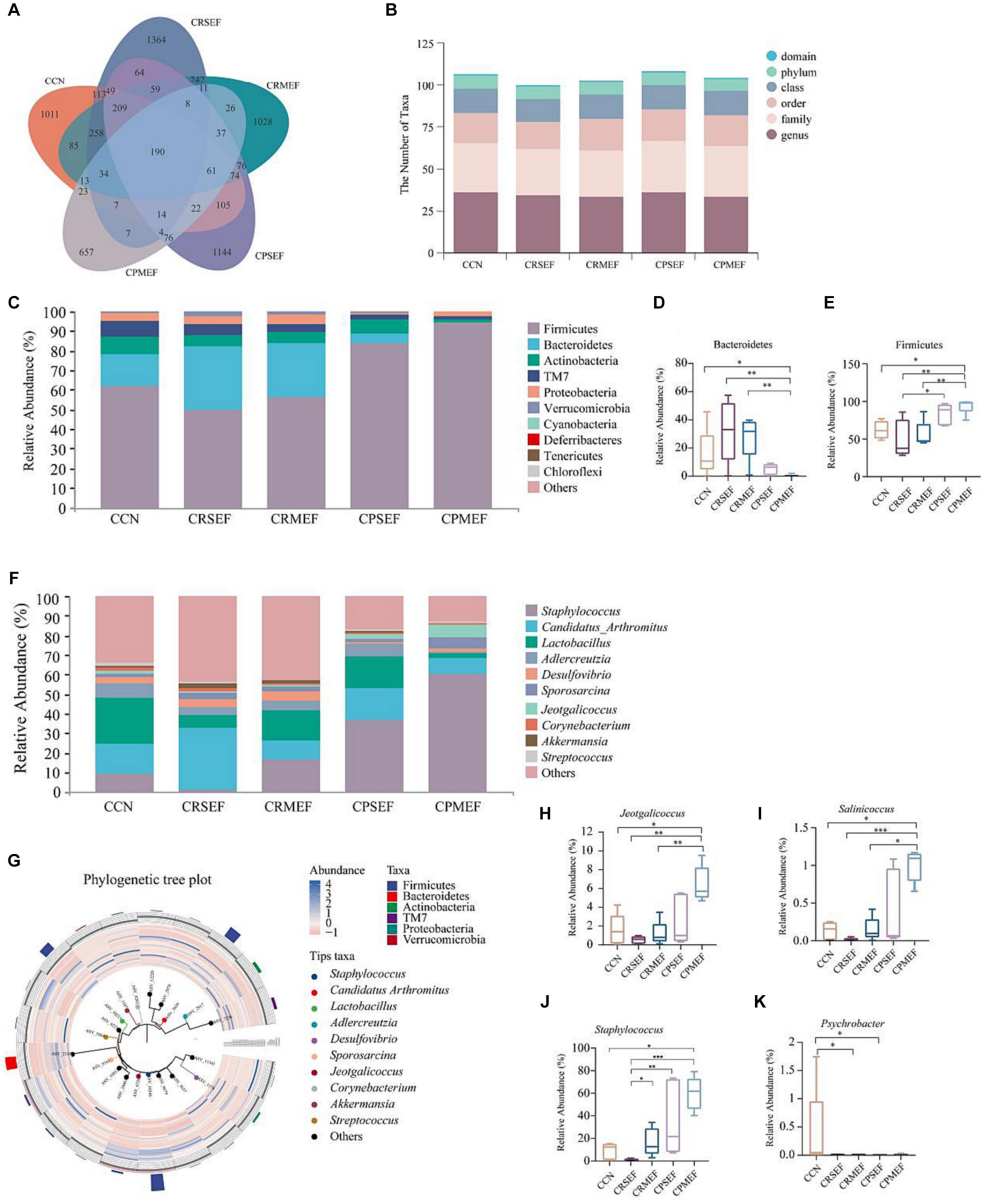
**(A)** ASVs Venn diagram. **(B)** Number of classification units in each group. **(C)** Composition of the intestinal contents microbiota at the phylum level. **(D)** Relative abundance of groups of Bacteroidetes. **(E)** Relative abundance of groups of Firmicutes. **(F)** Composition of the intestinal contents microbiota at the genus level. **(G)** Phylogenetic tree plot. **(H)** Relative abundance of groups of *Jeotgalicoccus*. **(I)** Relative abundance of groups of *Salinicoccus*. **(J)** Relative abundance of groups of *Staphylococcus*. **(K)** Relative abundance of groups of *Psychrobacter*. CCN: control group, CRSEF: raw small-flowered EF group, CRMEF: raw medium-flowered EF group, CPSEF: processing small-flowered EF group, CPMEF: processing medium-flowered EF group. *p < 0.05, **p < 0.01, ***p < 0.001.

The CCN, CRSEF, CRMEF, CPSEF, and CPMEF groups had 1,011, 1,364, 1,028, 1,144, and 657 unique ASVs, respectively, with a total of 190 identical ASVs in the five experimental groups (Figure 5A). The analysis of intestinal microbiota composition within the group showed that the intestinal content microbiota of the six classification levels changed after the EF intervention (Figure 5B). We further analyzed the composition of the intestinal content microbiota at different taxonomic levels between groups. At the phylum level, the intestinal contents microbiota was dominated by Firmicutes, Proteobacteria, Bacteroidetes, TM7, and Actinobacteria, with Firmicutes accounting for the largest proportion (Figure 5C). Compared with the CCN, CRSEF, and CRMEF groups, the relative abundance of Bacteroidetes was significantly decreased in the CPMEF group, while the relative abundance of Firmicutes was significantly increased (*p* < 0.05, Figures 5D,E). Compared with the CRSEF group, the relative abundance of Firmicutes was significantly increased in the CPSEF group (*p* < 0.05, Figure 5E).

At the genus level, *Staphylococcus*, *Candidatus Arthromitus*, *Lactobacillus*, *Adlercreutzia*, *Desulfovibrio*, etc., were predominant, with *Staphylococcus* accounting for the largest proportion (Figure 5F). According to the Phylogenetic tree plot, we found that *Staphylococcus* and *Jeotgalicoccus* belonged to Firmicutes (Figure 5G). Compared with the CCN group, the relative abundance of *Jeotgalicoccus*, *Salinicoccus*, and *Staphylococcus* was significantly increased in the CPMEF group (*p* < 0.05, Figures 5H–J). Compared with the CPMEF group, the relative abundance of *Jeotgalicoccus* and *Salinicoccus*, was significantly lower in both CRSEF and CRMEF groups (*p* < 0.05, Figures 5H–J). The relative abundance of *Psychrobacter* was significantly lower in the CRMEF and CPSEF groups compared to the CCN group (*p* < 0.05, Figure 5K).

#### 4.3.3 Different EF altered the characteristic microbiota and potential functional genes of intestinal contents microbiota in mice

**Figure 6:**
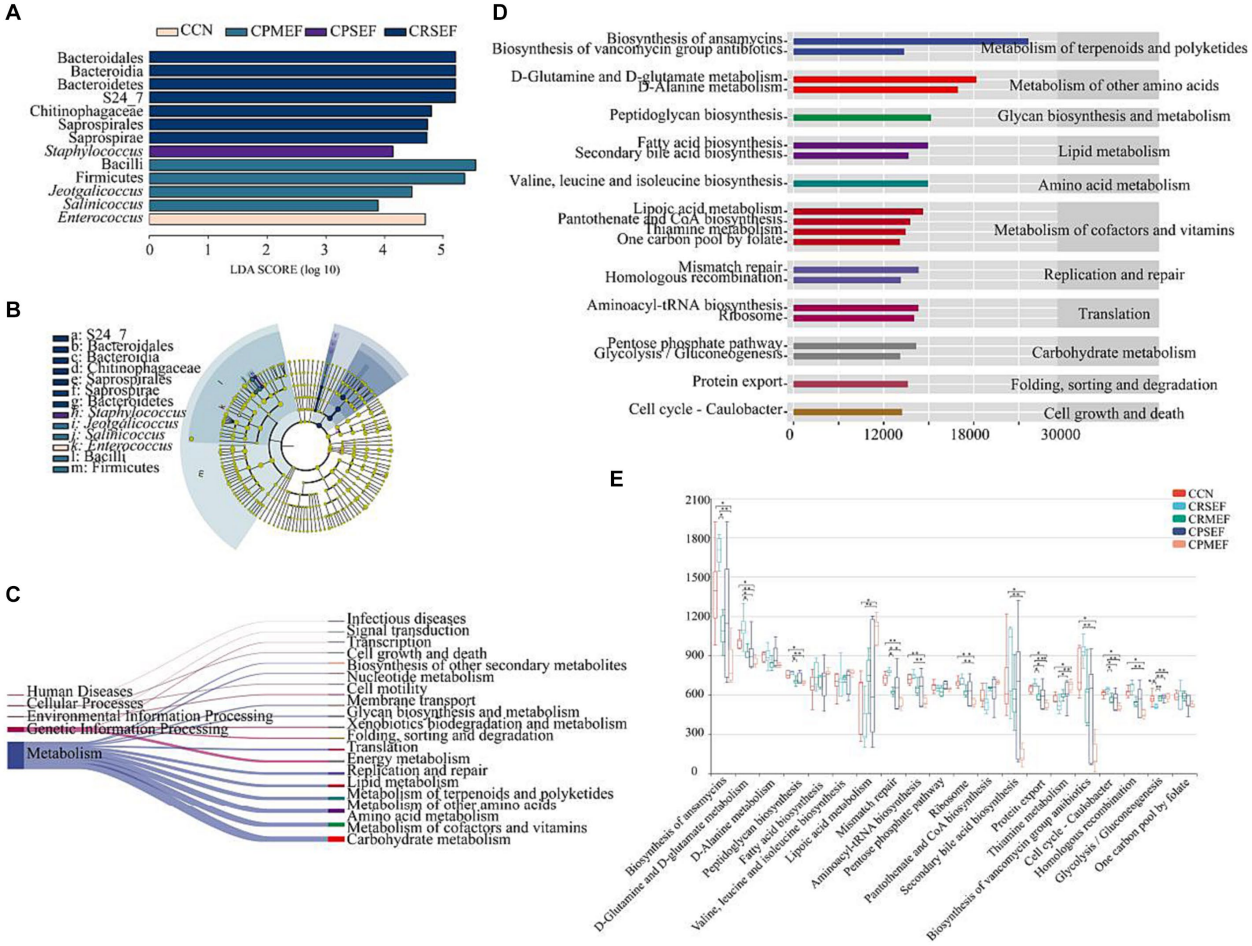
**(A)** Histogram of LDA to identify bacterial species with different levels. **(B)** The cladogram generated from the LEfSe analysis indicates the phylogenetic distribution from phylum to species of the intestinal contents microbiota. **(C)** Prediction of metabolic function of intestinal contents microbiota based on PICRUSt2. The left side is the primary KEGG metabolic pathway, and the right side is the secondary KEGG metabolic pathway. The line thickness represents the relative abundance of the KEGG metabolic pathway. **(D)** Prediction of metabolic function of intestinal contents microbiota based on PICRUSt2. The ordinate shows the classification of KEGG functional pathways: the second-order KEGG metabolic pathway on the left, the third-order KEGG metabolic pathway on the right, and the abscissa shows the abundance of KEGG functional pathways. **(E)** The difference box diagram of the third-order KEGG metabolic pathway in each group’s top 20 relative abundances. CCN: control group, CRSEF: raw small-flowered EF group, CRMEF: raw medium-flowered EF group, CPSEF: processing small-flowered EF group, CPMEF: processing medium-flowered EF group. *p < 0.05, **p < 0.01, ***p < 0.001.

Based on LEfSe analysis, we identified the characteristic bacteria with different changes in each group. When the LDA score was greater than 3, there were differences in the microbiota abundance of CCN, CPSEF, CPMEF, and CRSEF groups, among which 13 bacteria were identified as key discriminators. At the genus level, *Enterococcus* is characteristic of the CCN group, *Staphylococcus* is characteristic of the CPSEF group, and *Jeotgalicoccus*, *Salinicoccus* is characteristic of the CPMEF group (Figures 6A,B). Based on the KEGG database (Kanehisa and Goto, 2000; Kanehisa, 2019; Kanehisa et al., 2023), we predicted the metabolic pathways related to intestinal contents microbiota by PICRUSt2 analysis and further determined the effect of EF feeding on the potential metabolic function of intestinal contents microbiota in mice. We screened the top 20 metabolic pathways in relative abundance, mainly Cellular Processes, Environmental Information Processing, Genetic Information Processing, and Human Diseases, with the highest abundance of the Metabolism pathway (Figure 6C).

We further analyzed the top 20 level 3 metabolic pathways in terms of relative abundance. The results showed that the potential metabolic functions of the mouse intestinal contents microbiota were dominated by the Biosynthesis of ansamycins, D-Glutamine and D-glutamate metabolism, D-Alanine metabolism, Peptidoglycan biosynthesis Fatty acid biosynthesis was predominant (Figure 6D). Compared to the CCN group, the relative abundance of Biosynthesis of ansamycins, D-Glutamine and D-glutamate metabolism, Peptidoglycan biosynthesis, Mismatch repair, Aminoacyl-tRNA biosynthesis, Ribosome, Secondary bile acid biosynthesis, Protein export, Biosynthesis of vancomycin group antibiotics, Cell cycle - Caulobacter and Homologous recombination were significantly decreased in the CRMEF group. The relative abundance of Thiamine metabolism was significantly increased (*p* < 0.05, Figure 6E).

### 4.4 Correlation analysis of the characteristic microbiota of intestinal contents microbiota with liver and kidney function and potential functional genes

**Figure 7:**
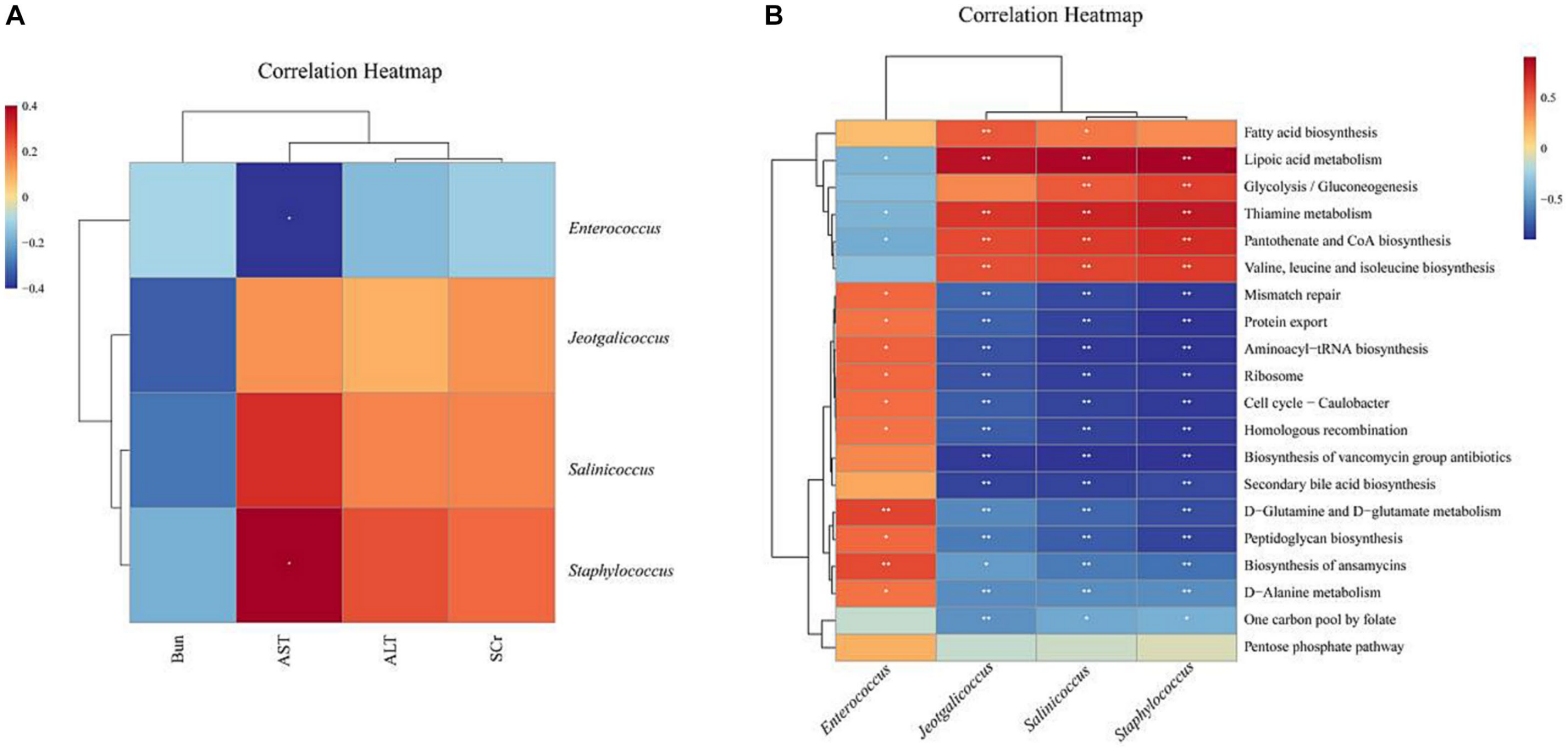
Heat map of correlation analysis: Blue represents a negative correlation, red represents a positive correlation, the closer the color is to blue, the stronger the negative correlation between the two parameters, the closer the color is to red, the stronger the positive correlation between the two parameters. **(A)** Heat map of correlation between characteristic microbiota of intestinal contents microbiota and liver and kidney function **(B)** Heat map of correlation between characteristic microbiota of intestinal contents microbiota and potential functional genes. *p < 0.05, **p < 0.01, ***p < 0.001.

Based on the above analysis, we found that EF affects liver and kidney function and can regulate intestinal microbiota dysbiosis. We further used correlation analysis to explore the correlation between altered intestinal microbiota, liver and kidney function, and potentially functional genes. AST was significantly negatively correlated with *Enterococcus* and positively correlated with *Staphylococcus*. AST, ALT, and SCr were positively correlated with *Staphylococcus*, *Jeotgalicoccus,* and *Salinicoccus*, but negatively correlated with *Enterococcus* (Figure 7A). *Staphylococcus*, *Jeotgalicoccus,* and *Salinicoccus* were significantly positively correlated with lipoic acid metabolism, and thiamine metabolism, and negatively correlated with Biosynthesis of ansamycins, D-Glutamine and D-glutamate metabolism, Peptidoglycan biosynthesis, Mismatch repair, Aminoacyl-tRNA biosynthesis, Ribosome, Secondary bile acid biosynthesis, Protein export, Biosynthesis of vancomycin group antibiotics, Cell cycle - Caulobacter, Homologous recombination. *Enterococcus*, on the other hand, was the opposite (Figure 7B).

## 5 Discussion

### 5.1 Effects of processing factors on EF liver and kidney toxicity

Drug-related liver and kidney injury is mainly due to the potential toxicity of drugs excreted by the liver and kidneys and their metabolites, which directly or indirectly cause damage to the organs, resulting in liver and kidney function and/or structural disorders. Drugs and their metabolites are mostly excreted from the urine through glomerular filtration and renal tubule secretion, so the kidneys are particularly vulnerable to the toxic effects of drugs (European Association for the Study of the Liver, 2019; Dobrek, 2023). Aristolochic acid and alkaloid compounds in some of these herbs are known to cause nephrotoxicity (Yang B. et al., 2018). The chemical composition of EF varies from size to size and from processing to processing, which may have an impact on the toxicity of EF. In this study, the content of limonin decreased significantly after processing of EF, EVO in PSEF decreased significantly and EVO in PMEF increased significantly. Studies have confirmed that EVO and its derivatives enhance thermogenesis and lipid oxidation and play a role in weight management and therapy (Gavaraskar et al., 2015). Limonin can reduce the weight of mice fed with high fat (Wang S. W. et al., 2022). In this study, it was found that the weight of mice after different EF feeding showed a decreasing trend, but the difference among groups was not significant, which may be related to the different contents of EVO and limonin.

Scr and Bun are metabolites in the body, mainly produced by muscle metabolism and cleared by the kidneys, and increased concentrations of Scr and Bun reflect decreased renal function, so the measurement of Scr and Bun can determine changes in renal function (Zhao et al., 2022; Hong et al., 2023). This study showed that Scr showed an increasing trend after feeding by different EF, Bun significantly increased in RMEF groups, and Bun significantly decreased in the PMEF group. However, due to the high metabolic capacity of the kidney, the Scr concentration generally does not increase in the early stage of kidney injury and only increases significantly when the glomerular filtration function is seriously reduced (Hong et al., 2023). Considering the metabolic characteristics of Scr, combined with histopathological sections, RMEF may have certain nephrotoxicity, and licorice processing can reduce nephrotoxicity. However, in this study, the kidney injury was not obvious, which may be due to insufficient feeding time or too low concentration. In the early stage of kidney injury, the glomeruli were not involved. In this study, AST significantly increased in the RMEF and PMEF groups. Although EVO is hepatocyte toxic, the mechanisms of its toxicity may involve lipid peroxidation damage, apoptosis, and cholestasis (Li et al., 2019; Gao et al., 2021). However, ALT mainly exists in hepatocytes and is the most sensitive detection index of liver function damage. AST usually exists in the mitochondria of cardiomyocytes and hepatocytes, with the highest content in myocardium, followed by the liver. AST will increase only when liver tissue is seriously damaged (Kalas et al., 2021; Lala et al., 2023). In combination with tissue sections, liver tissue sections in the PMEF group showed no obvious damage and ALT changes were not significant. Thus, elevated AST here may be a non-hepatic injury (hemolysis, myopathy, thyroid disease, exercise) rather than an EVO induced liver injury.

### 5.2 The influence of processing factors on EF regulation of intestinal microbiota

Intestinal microbiota directly or indirectly affects the metabolism and efficacy of many drugs, leading to drug activation, drug inactivation, or toxicity (Abdelsalam et al., 2020). The large microbial community in the intestine plays an important and complex role in drug absorption and health status of the liver. Most natural herbs, including EF, are administered orally, and intestinal microbiota first contacts the drug ingredients after entering the intestine (Džidić-Krivić et al., 2023). Intestinal ecological imbalance can indirectly or directly regulate the progress of renal diseases, among which the changes in the abundance and proportion of intestinal Bacteroidetes and Firmicutes can reflect renal diseases. Herbal alleviates renal diseases by regulating intestinal microbiota, which can reduce Bacteroidetes and increase Firmicutes, showing a direct correlation with renal function parameters (Chen et al., 2021; Saranya and Viswanathan, 2023). This study showed that after the processing of EF feeding, the diversity of Bacteroidetes in the intestinal contents of mice decreased, and Firmicutes increased significantly, while renal toxicity decreased significantly. Therefore, we hypothesize that the attenuated PEF nephrotoxicity may be associated with a significant decrease in Bacteroidetes and a significant increase in Firmicutes.

Staphylococcaceae covers a large group of Gram-positive bacteria, including *Jeotgalicoccus*, *Macrococci*, *Nosocomicocus*, *Salinicoccus,* and *Staphylococcus*, which are considered the pathogens of several serious diseases (Tang et al., 2015). *Staphylococcus* is a common pathogenic bacterium, the most common of which is *Staphylococcus aureus*, that promotes inflammation leading to infectious threats to global public health and is a major pathogen causing a range of diseases (Chand et al., 2023; Subramanian and Natarajan, 2023) *Psychrobacter* is a kind of bacteria resistant to low temperature, dryness, and starvation pressure, and it grows in low temperature by down-regulating energy metabolism (Kim et al., 2012). This study found that *Staphylococcus* was the characteristic bacteria of PSEF, and *Jeotgalicoccus,* and *Salinicoccus* were the characteristic bacteria of PMEF. In this study, CPMEF significantly decreased the diversity of intestinal microbiota and increased *Staphylococcus*, *Jeotgalicoccus,* and *Salinicoccus*. Compared with the CPMEF group, CRMEF significantly increased the diversity of intestinal microbiota and significantly decreased the relative abundance of *Staphylococcus*, *Jeotgalicoccus,* and *Salinicoccus*. Studies have found that limonin can significantly increase the richness of colon microbiota, significantly inhibit bacterial toxins and *Staphylococcus aureus* infection markers, improve intestinal injury and ulcers, and have a protective effect on the intestine (Gu et al., 2019; Jia et al., 2023). Some studies have found that adenine induced damage extends beyond the kidneys to affect the intestine as well, and the intestinal microbiota is a key mediator of the chemical’s toxicological effects. The correlation between chemical toxicity, intestinal microbiota, and organ-specific damage reveals the complex interaction between chemical toxicity and intestinal microbiota (Li et al., 2023). On the one hand, we speculate that EF components may have toxic effects on specific microbiota, leading to the reduction or proliferation of microbiota, for example, the RMEF group has a higher limonin content, and compared with the PMEF group, the intestinal harmful bacteria are reduced and their diversity is increased. Therefore, REF increased microbial diversity and decreased harmful bacteria, which may be related to higher limonin content. On the other hand, changes in intestinal microbiota may affect the metabolism and absorption of EF in the intestine, thereby regulating its toxicity. For example, the increase of harmful bacteria in the PMEF group may affect intestinal mucosal permeability, and thus the physiological and metabolic processes of EF.

### 5.3 Effect of processing factors on potential functional genes of intestinal contents microbiota

The liver plays a key role in the homeostasis of most amino acids and determines whether specific amino acids are absorbed by the liver or circulated throughout the body, whereas hepatic amino acids are metabolic substrates for a variety of essential compounds (Paulusma et al., 2022). Disturbances in amino acid metabolism are associated with the progression of hepatotoxicity. The amino acid metabolic pathways, metabolic pathways of energy, glutathione, and porphyrin are downregulated after liver injury, and *Staphylococcus* abundance increases and is associated with liver injury (Wang C. et al., 2022). SCFAs, bile acids, and fatty acids are common metabolites of the microbiome that regulate intestinal permeability, glucose and lipid metabolism, the immune system, and inflammatory responses (Xiao et al., 2021; Min et al., 2022). Glutamate is a key hepatic amino acid metabolite produced through transamination during the catabolism of several amino acids (Pfennig et al., 2013).

This study showed that after RMEF feeding, D-Glutamine and D-glutamate metabolism, Peptidoglycan biosynthesis, Aminoacyl-tRNA biosynthesis, Secondary bile acid biosynthesis, Protein export, Biosynthesis of antibiotics were significantly reduced. It is negatively correlated with *Staphylococcus*, *Jeotgalicoccus,* and *Salinicoccus*, while Lipoic acid metabolism and Thiamine metabolism, AST, ALT, and Scr were positively correlated with *Staphylococcus*, *Jeotgalicoccus*, and *Salinicoccus*. The results indicated that the changes in intestinal microbiota after EF feeding may affect the amino acid metabolism of hepatocytes and regulate the changes of related liver amino acid metabolism pathway, among which the abundance of *Staphylococcus*, *Jeotgalicoccus,* and *Salinicoccu* are closely related to liver function. Liver injury was not obvious in this study, but the results of bacterial microbiota showed that the increase of harmful bacteria was correlated with the decrease in liver metabolic function. Therefore, EF may have some liver damage too, but the toxicity concentration was not determined in this study for the time being because the concentration gradient and time gradient were not set up in this study, which is a shortcoming of the study.

## 6 Conclusion

In conclusion, PMEF significantly increased harmful bacteria (*Staphylococcus*, *Jeotgalicoccus,* and *Salinicoccu*) and decreased beneficial bacteria. SEF with 5 times the clinical dose showed nephrotoxicity, and SEF nephrotoxicity decreased after processing, but EF hepatotoxicity was not significant, which may be due to insufficient dose concentration and time.

## Data availability statement

The datasets presented in this study can be found in online repositories. The names of the repository/repositories and accession number(s) can be found at: NCBI - PRJNA976505.

## Ethics statement

The animal studies were approved by Animal Experimentation Ethics Committee of the Hunan University of Chinese Medicine. The studies were conducted in accordance with the local legislation and institutional requirements. Written informed consent was obtained from the owners for the participation of their animals in this study.

## Author contributions

XL: Conceptualization, Investigation, Writing – original draft, Funding acquisition. JL: Writing – original draft, Conceptualization, Data curation, Formal analysis, Investigation. JD: Conceptualization, Methodology, Project administration, Validation, Writing – original draft. NX: Methodology, Software, Conceptualization, Formal analysis, Writing – original draft. YP: Conceptualization, Funding acquisition, Formal analysis, Writing – original draft. QT: Conceptualization, Funding acquisition, Writing – review & editing, Project administration. LC: Conceptualization, Funding acquisition, Visualization, Methodology, Writing – original draft.

## Footnotes

^1^https://www.kegg.jp/

^2^https://www.omicstudio.cn/tool
